# A novel decellularized nerve graft for repairing peripheral nerve long gap injury in the rat

**DOI:** 10.1007/s00441-022-03682-1

**Published:** 2022-09-17

**Authors:** Estefanía Contreras, Sara Bolívar, Núria Nieto-Nicolau, Oscar Fariñas, Patrícia López-Chicón, Xavier Navarro, Esther Udina

**Affiliations:** 1 Department of Cell Biology, Physiology and Immunology, Institute of Neuroscience, Universitat Autònoma de Barcelona, and CIBERNED, ISCIII, 08913 Bellaterra, Spain; 2 Barcelona Tissue Bank, Banc de Sang I Teixits (BST), Barcelona, Spain; 3 Biomedical Research Institute (IIB-Sant Pau; SGR1113), Barcelona, Spain

**Keywords:** Nerve regeneration, Decellularization, Nerve allograft, Nerve injury

## Abstract

Decellularized nerve allografts are an alternative to autograft for repairing severe nerve injuries, since they have higher availability and do not induce rejection. In this study, we have assessed the regenerative potential of a novel decellularization protocol for human and rat nerves for repairing nerve resections, compared to the gold standard autograft. A 15-mm gap in the sciatic nerve was repaired with decellularized rat allograft (DC-RA), decellularized human xenograft (DC-HX), or fresh autograft (AG). Electrophysiology tests were performed monthly to evaluate muscle reinnervation, whereas histological and immunohistochemical analyses of the grafts were evaluated at 4 months. A short-term study was also performed to compare the differences between the two decellularized grafts (DC-RA and DC-HX) in early phases of regeneration. The decellularization process eliminated cellularity while preserving the ECM and endoneurial tubules of both rat and human nerves. Higher amount of reinnervation was observed in the AG group compared to the DC-RA group, while only half of the animals of the DC-HX showed distal muscle reinnervation. The density of myelinated axons was significantly higher in AG compared to both DC grafts, being this density significantly higher in DC-RA than in DC-HX. At short term, fibroblasts repopulated the DC-RA graft, supporting regenerated axons, whereas an important fibrotic reaction was observed around DC-HX grafts. In conclusion, the decellularized allograft sustained regeneration through a long gap in the rat although at a slower rate compared to the ideal autograft, whereas regeneration was limited or even failed when using a decellularized xenograft.

## Introduction

Peripheral nerve injury results in partial or total loss of the sensory and motor functions dependent on the injured nerve(s), with important consequences for the quality of life of affected subjects (Navarro et al. 2007). It is estimated an incidence of nerve injuries of 13.9/100,000 inhabitants per year (Asplund et al. 2009) resulting in more than 300,000 such injuries in the EU per year. Although peripheral neurons have the ability to regenerate their axons and eventually reinnervate the previously denervated target organs, clinical and experimental evidence shows that regeneration is often unsatisfactory, especially following severe injuries (Pfister et al. 2011; Lovati et al. 2018).

Surgical repair is needed after nerve transections, to re-unite the two nerve stumps, with the aim to facilitate proximal axons to regenerate through the distal degenerating nerve and to finally reinnervate their target organs (Pfister et al. 2011). However, direct suture is not always possible, and long nerve gaps must be bridged. The clinical gold standard repair technic in clinics is the interposition of an autologous nerve graft between the proximal and the distal stump (Grinsell and Keating 2014; Gaudin et al. 2016; Kornfeld et al. 2021). Although it sustains nerve regeneration across long defects, the use of an autografts has some disadvantages, such as limited availability of donor nerves, increased operating time, and morbidity at the site of extraction (e.g., pain, scars, neuroma, and sensory loss).

Although devoid of the drawbacks of autografts, allografts induce an immune rejection response by the recipient body and therefore require systemic immunosuppressive therapy. The most immunogenic elements in the allografts are Schwann cells and myelin sheaths, since their membrane present major histocompatibility complex antigens (Evans et al. 1995). Immune compatibility between donor and host is thus important to guarantee axonal regeneration, in both nerve grafts and artificial guides pre-filled with transplanted Schwann cells (Rodríguez et al. 2000). A promising alternative to autografts is a decellularized nerve allograft (Hundepool et al. 2017; Lovati et al. 2018; Philips et al. 2018). The decellularization involves several processes that guarantee immunogenic free scaffolds from native nerves, while preserving the extracellular matrix (ECM) components and the biomechanical properties of the grafts (Szynkaruk et al. 2013; Nieto-Nicolau et al. 2021). Therefore, decellularization of human peripheral nerves is an innovative strategy in tissue engineering and it can become an alternative to autografts to repair long-gap peripheral nerve injuries. For its clinical translation, it is mandatory to evaluate the pro-regenerative potential of optimized protocols of decellularization of human cadaver nerves. However, the preclinical evaluation of human grafts in experimental models has an important limitation, since these grafts become xenografts when transplanted to experimental animals, and the immune rejection caused by donor antigens (Udina et al. 2003) or other inter-species differences (Wood et al. 2014) can interfere with their regenerative potential. Nevertheless, it is worth to further explore the potential of decellularized xenografts as a repair alternative to grafts from the same species origin. Therefore, in this study, we aimed to perform a comparative assessment of the effective axonal regeneration, along a critical nerve gap of 15 mm length induced in the sciatic nerve of adult rats, repaired with a decellularized allograft, a decellularized human xenograft, or the gold standard autograft.

## Materials and methods

### Ethics approval

This study followed the ethical precepts of the Declaration of Helsinki and was approved by local ethics committee. Human tissue was processed according to guidance for clinical use (EEC regulations 2004/23/CE and 2006/17/CE) and to the legal requirements for the use of biological samples for research in Spain (Law 14/2007 and RD 1716/2011). Ethical Committee approval was issued by CEIm Hospital Valle Hebron, Barcelona; PR (BST) 314/2019. In all cases, informed consent was obtained from the donors’ relatives.

Regarding animal experiments, all the procedures were approved by the Ethics Committee of the *Universitat Autònoma de Barcelona* and *Generalitat de Catalunya* (reference #10,306) and followed the European Community Council Directive 2010/63/EU of the European Parliament on the protection of animals used for scientific purposes. In addition, we followed the ARRIVE guidelines and committed ourselves to the 3Rs of laboratory animal research.

### Preparation of acellular nerve grafts

Human sural nerves from eleven deceased donors were obtained from the Barcelona Tissue Bank-Banc de Sang i Teixits (BTB-BST, Barcelona, Spain; https://www.bancsang.net/) within 24 h post-mortem. Donor screening included, but may not be limited to, the review of complete social and medical history, physical examination of the donor, complete serological and microbiological testing during retrieval, histopathological analysis, and any other information pertaining to risk factors for relevant communicable diseases. Sural nerve fragments were retrieved from the donor and were placed into a sterile container with Roswell Park Memorial Institute (RPMI) media without phenol (Gibco, Carlsbad, CA, USA) plus 1% antibiotics (vancomycin [Pfizer, MA, Spain], penicillin [Normon, MA, Spain], and streptomycin [Reig Jofre, BCN, Spain]) at 4 °C until its decellularization in a clean room environment.

Rat sciatic nerves were harvested from Sprague–Dawley donor rats, under deep anesthesia and with sterile conditions. Samples were placed in sterile phosphate buffered solution (PBS) with antibiotic/antifungal agents (Sigma-Aldrich) and processed within 24 h.

The decellularization protocol for human nerve samples was as described previously (Nieto-Nicolau et al. 2022). To decellularize rat nerves, that protocol was adapted, reproducing sequential incubations and using zwitterionic and non-ionic detergents for 3 days. Zwitterionic sulfobetaines 10 and 16 were purchased from Sigma-Aldrich (Munich, Germany). Non-ionic Triton X-200 was changed by Triton X-100 (Sigma-Aldrich). This protocol was optimized adding an incubation with hypertonic 1 M NaCl (Sigma-Aldrich) during 4 h and 0.1 mg/ml Pulmozyme DNASE (Roche, Barcelona, Spain) for 3 h. After DNASE treatment, the nerves were washed once in 0.5 M Tris–EDTA buffer and several times in ultrapure water. The process was carried out at room temperature for 4 days.

#### Histological evaluation of decellularized nerve grafts

Decellularized nerve grafts were fixed in paraformaldehyde 4% for 24 h at 4 °C, then transferred to phosphate buffered (PB) and incubated with saccharose 30% for 24–48 h. Samples were cryo-embedded with Tissue Tek^®^ OCT compound (Sakura, Fleminweg, Netherlands), and cross sections of 15 µm thickness were obtained with a cryotome. After blocking with a solution of normal goat serum or normal donkey serum (10%) containing 0.3% Triton, sections were incubated overnight at 4 °C with primary antibodies against S-100 protein (S100; Schwann cells; 1:50; 22,520-DiaSorin), neurofilament (NF200; myelinated axons; 1:400; AB5539-Millipore), non-collagenous connective tissue glycoprotein (laminin; 1:500; AHP420-Biorad), and collagen (Collagen IV; 1:400; 134,001-Setareh Biotec). Following washes, sections were incubated with secondary antibodies bound to Alexa Fluor 488 and Alexa Fluor 594. Sections were also stained with DAPI (DAPI; nuclei; 1:100; D9564-10MG-Sigma) and myelin stain (Fluoromyelin; myelin; 1:300; F34651-Invitrogen). Immunolabeled sections were viewed under epifluorescence microscopy (Olympus BX51).

### In vivo long-term studies

Female Sprague–Dawley rats (12 weeks of age and weighing 296–322 g) were used. They were housed under a 12-h-light–dark cycle in a temperature-controlled animal care facility with ad libitum access to water and food.

Seventeen female Sprague–Dawley rats were randomized into three different groups: autograft (AG) (*n* = 6), decellularized rat allograft (DC-RA) (*n* = 5), and decellularized human xenograft (DC-HX) (*n* = 6), and followed for 120 days. Rats were anesthetized with ketamine (75 mg/kg) and medetomidine (0.01 mg/kg) intraperitoneally. The right hindlimb was shaved and sterilized with povidone-iodine solution. A skin incision was made, and the right sciatic nerve was exposed and resected. In the autograft group, a 15-mm-long nerve segment of the sciatic nerve was excised and then, sutured again in place using three 10–0 nylon epineural sutures. In the rat allograft and human xenograft, a 15-mm decellularized rat allograft or a 15-mm decellularized human xenograft was sutured to the host nerve stumps. The muscles were sutured with 6–0 and the skin with 3–0 silk and staples. Postoperative care included amitriptyline (20 ml/l) in drinking water to prevent autotomy (Navarro et al. 1994) and buprenorphine 0.03 mg/kg subcutaneously to treat postoperative pain.

#### Electrophysiological tests

Reinnervation of target muscles was assessed at 30, 60, 90, and 120 days post-injury (dpi) by motor nerve conduction tests. Animals were anesthetized as indicated above, and the sciatic nerve was stimulated with transcutaneous needle electrodes placed at the sciatic notch delivering single pulses of increasing intensity (Synergy Medelec, Viasys HealthCare), and the compound muscle action potential (CMAP) was recorded by placing electrodes on the tibialis anterior (TA), gastrocnemius (GA), and plantar interosseus (PL) muscles. The reference electrode was placed at the fourth toe, and a ground electrode was placed at the knee. The amplitude and latency of the CMAP were measured. The contralateral intact limbs were used as control. The rat body temperature was maintained throughout the test with a thermostatic warming flat coil.

#### Histological evaluation

At the end of the 120-day follow-up, animals were euthanized by an intraperitoneal injection of pentobarbital, transcardially perfused with 4% paraformaldehyde in PBS for 30 min, and the sciatic nerves were collected and divided in three parts.

The middle segment of the nerve graft was processed for immunofluorescence labeling as indicated above. Serial sections were incubated for staining axons (NF200), Schwann cells (S100), ionized calcium-binding adapter molecule 1 (Iba1; macrophages; 1:500; 19–19,741-Rafer), and laminin. Samples were washed and incubated with secondary antibodies Alexa Fluor 488 goat anti-chicken (1:200; A11039-Invitrogen), Alexa Fluor 488 donkey anti-goat (1:200; A11055-Invitrogen), and Alexa Fluor 594 goat anti-rabbit (1:200; A21207-Invitrogen) diluted in PBS-Triton 0.3%.

The proximal and distal segments of the grafted nerve, including suture levels, were post-fixed in 3% glutaraldehyde −3% paraformaldehyde in cacodylate-buffer solution (0.1 m, pH 7.4) at 4 °C. These segments were post-fixed with osmium tetroxide (2%, 2 h) and dehydrated through ethanol series prior to embedding in epon resin. Semithin 0.5 μm thick sections were stained with toluidine blue and examined by light microscopy (Olympus BX40). Sets of images obtained at 1000 × were chosen by systematic sampling of squares representing at least 30% of the nerve cross-sectional area (Gómez et al. 1996). Measurements of cross-sectional area of the whole nerve and counts of the number of myelinated nerve fibers were carried out using ImageJ software.

### In vivo short-term studies

Twelve female Sprague–Dawley rats were randomized in two experimental groups of 6 animals each, according to the repair procedure: DC-RA and DC-HX. In each group, half of the animals were assessed at 7 days and half at 14 days post-injury. Surgeries were performed as described above. However, in this case, a segment of the sciatic nerve was excised and substituted by a 12-mm DC-RA or 12-mm DC-HX, therefore using a shorter graft than for the long-term study. The proximal and distal nerve stumps were sutured by 10–0 nylon epineural sutures.

#### Histological evaluation

At the end of the follow-up (7 or 14 dpi), animals were euthanized and perfused as described. The sciatic nerve was harvested and divided in two equal parts, stored in cryoprotective solution of PBS-sucrose 30% at 4 °C for 24 h. The proximal nerve segment at 7 dpi and the proximal and distal nerve segment at 14 dpi were embedded in Tissue Tek and 15-μm-thick longitudinal sections cut serially. For immunostaining, sections were incubated overnight with primary antibodies for staining axons (NF200), Schwann cells (S100), macrophages (Iba1), and fibroblasts (vimentin; 1:400; ab92547-Abcam). Samples were washed and incubated with secondary antibodies as above. Finally, sections were washed and cover-slipped with Fluoromount containing DAPI (1:10,000, Sigma) for nuclear counterstain.

### In vitro study

#### Dissociate culture of DRG

Nine adult C57BL/6 J mice were euthanized, and DRGs were dissected. Dissociation and culture of DRG cells were performed as previously described (Alé et al. 2014). Briefly, the ganglia were cleaned under a dissecting microscope and enzymatically digested by incubation for 30 min at 37 °C in 10% trypsin, 10% collagenase, and 10% DNAse. The enzymatic activity was halted by adding 1 ml of DF10S, and tissue suspension was centrifuged at 900 g for 3 min. Then, the pellet was resuspended in 1 mL of culture medium and mechanically dissociated.

A total of 12,000 cells were plated on coverslips coated with poly-D-lysine plus human laminin (1 µg/µl, Sigma-Aldrich) or mice laminin (1 µg/µl, Sigma-Aldrich). After 24 h in culture, cells were fixed with 4% paraformaldehyde for 20 min, and neurons were immunostained with β3-tubuline antibody (1:500, MMS-435P/801202, Biolegend), NF200 antibody (1:800) and DAPI (1:1000, D9564-10MG-Sigma). The length of the longest neurite from β3-tubuline and NF200 positive neurons was measured at 100 × magnification under epifluorescence microscopy. Three different cultures using both conditions in at least three different wells each were analyzed.

### Data analysis

Data are expressed as mean ± standard error of the mean (SEM). The results of functional tests and histology were analyzed by two-way ANOVA. The results of the longest neurites from DRG cultures were compared using unpaired *t*-test. Statistical analyses were made with GraphPad Prism 8 software. A *p* < 0.05 was considered as significant.

## Results

### Effectiveness of the decellularization procedure

**Fig. 1:**
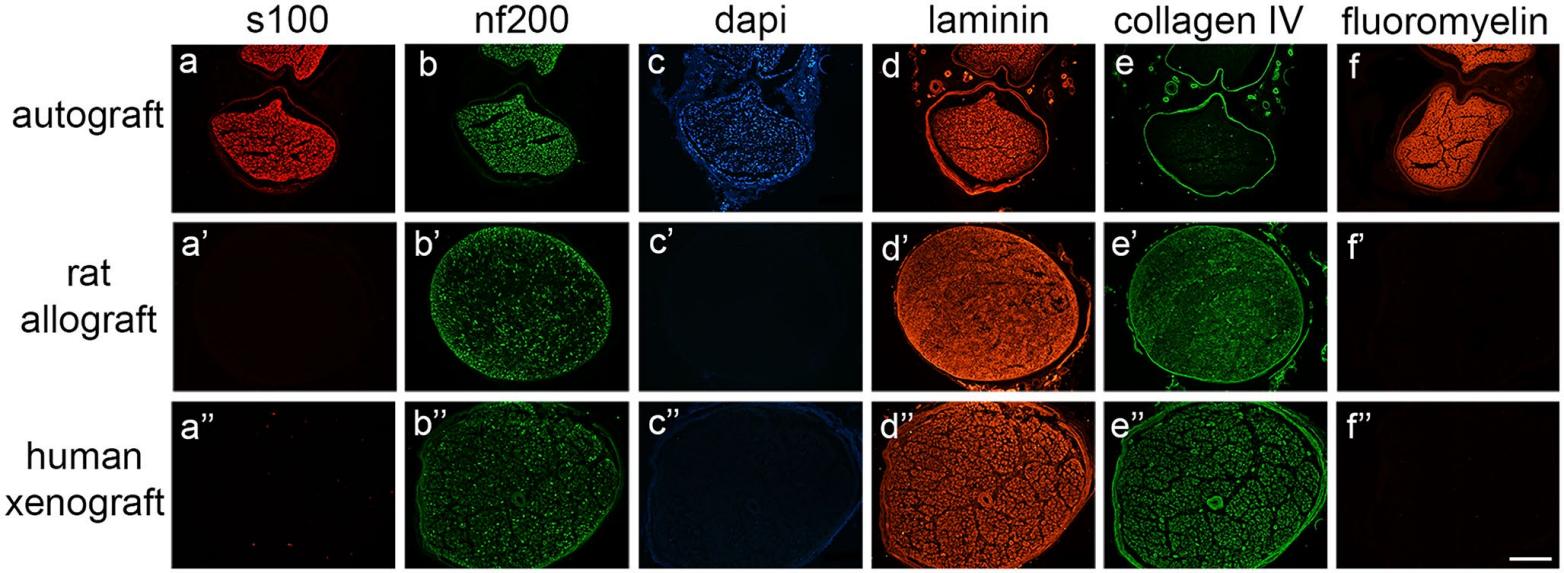
Decellularization efficiency. Representative micrographs showing immunolabeling of Schwann cells (s100) (**a″**), myelinated axons (nf200) (**b″**), nuclei (dapi) (**c″**), extracellular matrix proteins (laminin and collagen IV) (**d″** and **e″**, respectively), and myelin (fluoromyelin) (**f″**), in a control nerve (native nerve) (**a**, **b**, **c**, **d**, **e**, **f**), a decellularized rat nerve allograft (**a′**, **b′**, **c′**, **d′**, **e′**, **f′**) and a decellularized human nerve xenograft (**a″**, **b″**, **c″**, **d″**, **e″**, **f″**). Images taken at 100 × magnification; scale bar 400 µm

Cross sections of human and rat decellularized nerve grafts were processed for immunohistochemistry. Immunolabeling against laminin and collagen IV indicated that the structure of the extracellular matrix and endoneurial tubules was well preserved and comparable. Neurofilament remnants were present though their concentration was highly reduced and fragmented, compatible with degradation of axons, whereas the absence of DAPI and S100 staining indicated that cell contents were completely removed (Fig. 1).

### In vivo repair of a critical nerve gap with decellularized grafts

#### Electrophysiological test results

**Fig. 2:**
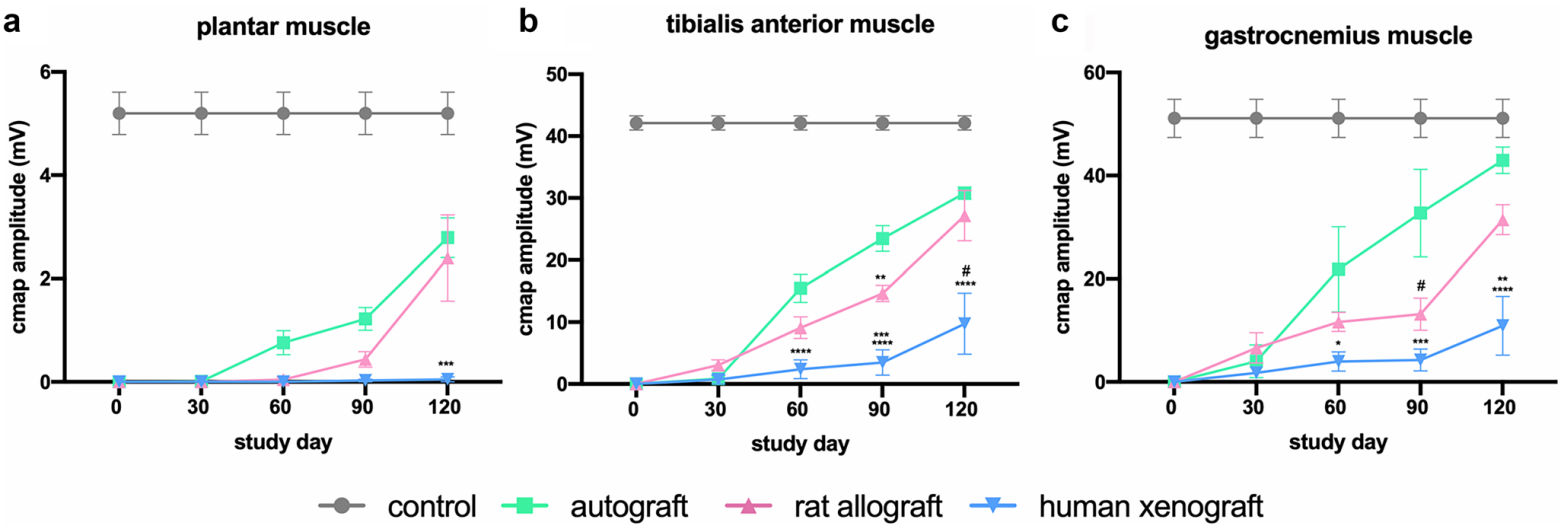
Electrophysiological evaluation of nerve regeneration along the 120-day follow-up after sciatic nerve section and repair. Results are presented as mean ± SEM. Statistical analysis was performed using 2-way ANOVA. Plots show the amplitude of CMAPs of plantar (***p < 0.001 vs AG), tibialis anterior (**p < 0.01 vs AG; ***p < 0.001 vs DC-RA; ****p < 0.0001 vs AG; #*p* < 0.0001 vs DC-RA) and gastrocnemius (*p < 0.5 vs AG; #*p* < 0.5 vs AG; **p < 0.01 vs DC-RA; ***p < 0.001 vs AG; ****p < 0.0001 vs AG) muscles

Nerve conduction tests were performed to assess reinnervation of TA, GM, and PL muscles (Fig. 2). At 60 dpi, all animals from AG and DC-RA groups showed evidence of reinnervation in the TA and GM muscles, whereas in the PL muscle, CMAPs were recorded in all animals of AG group but only in 20% of DC-RA group. In the DC-HX rats, only 50% of the animals showed evidence of reinnervation of the TA and GM muscles and none for the PL muscle. At 90 dpi, the amplitude of the CMAPs increased. All animals from AG and DC-RA groups had reinnervation of TA, GM, and PL muscles, whereas half of the animals of the DC-HX had positive values in TA and GM muscles and only one in the PL muscle. At the end of the follow-up (120 dpi), the mean CMAP amplitude of TA (30.8 ± 1.0 mV), GM (43.0 ± 2.6 mV), and PL (2.8 ± 0.4 mV) muscles in the AG group were higher than in the DC-RA group (23.1 ± 3.7 mV, 31.4 ± 2.9 mV, and 1.5 ± 0.2 mV, respectively). Animals from DC-HX group showed significantly lower values compared to AG and DC-RA groups in the TA (9.7 ± 4.9 mV; *p* < 0.0001), GM (10.9 ± 5.7 mV; *p* < 0.01 vs DC-RA, and *p* < 0.0001 vS AG) and PL muscles (0.05 ± 0.05 mV; *p* < 0.001 vs DC-RA and *p* < 0.0001 vs AG). Moreover, only half of the animals repaired with the human decellularized graft showed reinnervation signs in TA and GM muscles, and just 1 of the 6 animals in the PL muscle.

#### Histological nerve assessment

**Fig. 3:**
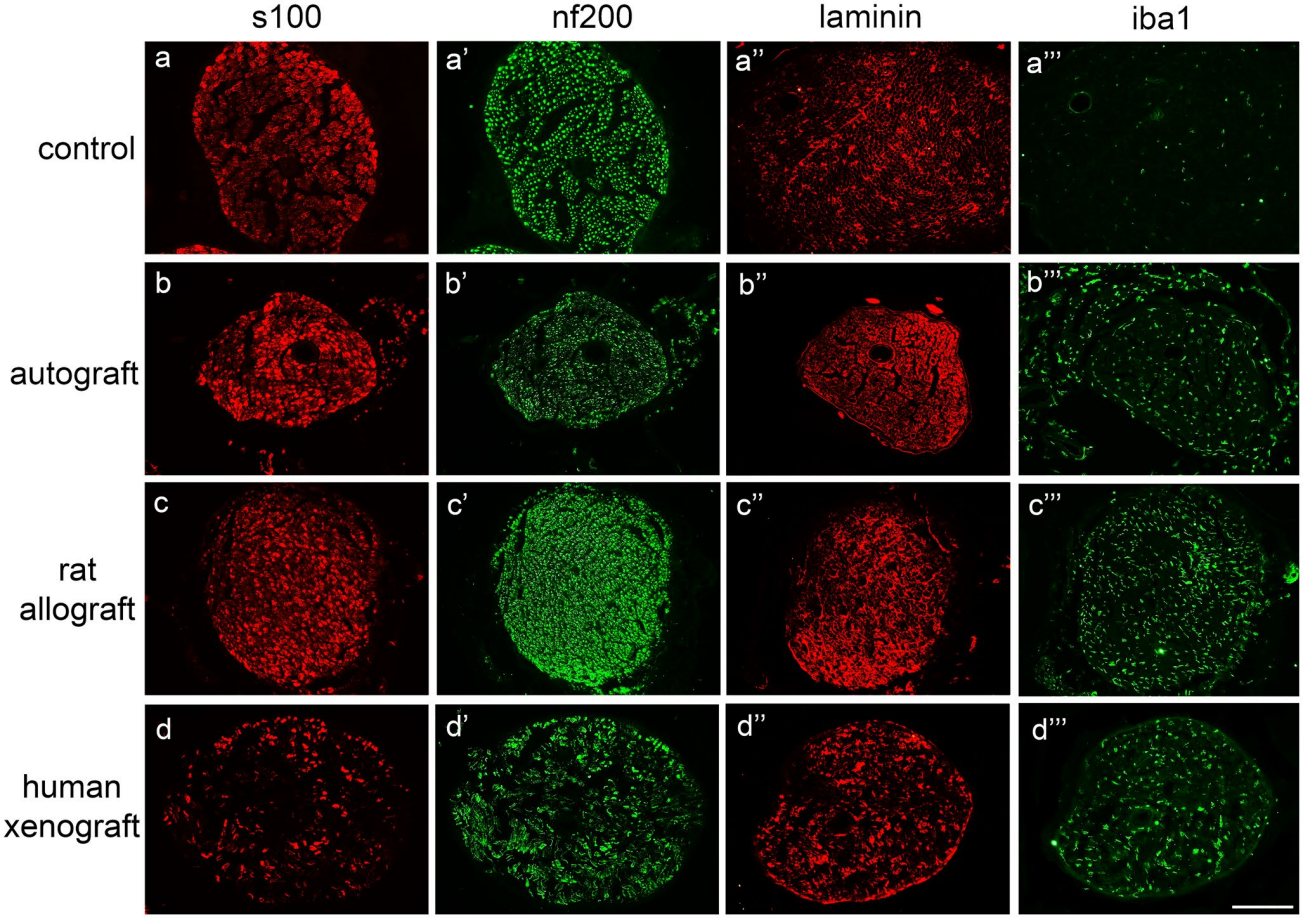
Immunohistochemical characterization of the grafts 120 days after implantation. Representative micrographs of nerve grafts harvested at 120 dpi showing immunofluorescence of Schwann cells labeled against S100 (**a**, **b**, **c**, **d**), myelinated axons labeled against neurofilament 200 (NF200) (**a′**, **b′**, **c′**, **d′**), extracellular matrix proteins labeled against laminin (**a″**, **b″**, **c″**, **d″**), and macrophages labeled against Iba1 (**a‴**, **b‴**, **c‴**, **d‴**) in cross sections of the graft in a control nerve (no decellularized native nerve) (**a‴**), autograft (**b‴**), rat allograft (**c‴**), and human xenograft (**d‴**) groups. Images taken at 200 × magnification; scale bar 150 µm

At the end of follow-up, at 120 dpi, nerves were harvested and processed for histological analysis. There were numerous regenerated myelinated axons and Schwann cells within the AG and DC-RA grafts, but quite limited in DC-HX grafts. The endoneurial basal lamina tubules were preserved in all the grafts, and the amount of macrophage immunolabeling was also similar (Fig. 3).

**Fig. 4:**
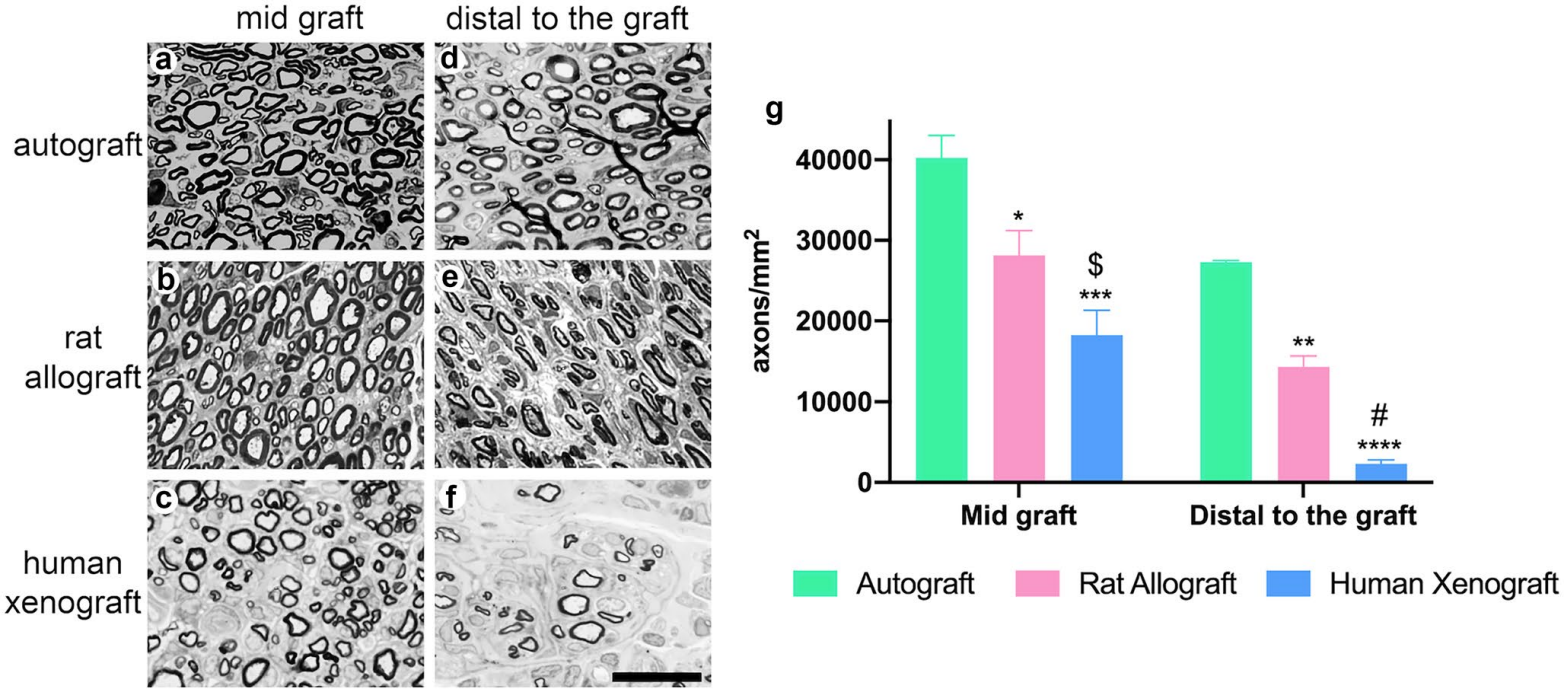
Histological evaluation of the regenerative potential of the grafts at 120 days. Representative transverse semithin sections of the mid graft (**a**, **b**, **c**) and distal to the graft (**d**, **e**, **f**) in autograft (**a**, **d**), decellularized rat allograft (**b**, **e**), and decellularized human xenograft (**c**, **f**) groups, stained with toluidine blue. Images were taken at × 1000 magnification; scale bar 10 μm. (**g**) Plots showing density of myelinated axons in the sciatic nerve at the mid graft and distal to the graft in the three groups. *p<0.5 vs AG; $*p*<0.5 vs DC-RA; **p<0.01 vs AG; #*p*<0.01 vs DC-RA; ***p<0.001 vs AG; ****p<0.0001 vs AG

Quantitative analysis demonstrated that the density of myelinated axons was statistically higher in AG (40267.78 ± 2775.7 axons/mm^2^) than in the DC-RA (28132.47 ± 3084.11 axons/mm^2^, *p<0.5 vs AG) and DC-HX (18244.98 ± 3070.41 axons/mm^2^, $*p*<0.5 vs DC-RA and ***p < 0.001 vs AG) groups in the middle of the graft. Distal to the graft, the myelinated fiber density was also significantly higher in AG group (27293.08 ± 195.8 axons/mm^2^) compared to DC-RA (14337.13 ± 1324.54 axons/mm^2^, **p < 0.01) and DC-HX (2289.29 ± 513.73 axons/mm^2^, #*p*<0.01 vs DC-RA and ****p < 0.0001). All animals showed regeneration in the middle of the grafts. Distal to the nerve graft, all animals from AG and DC-RA had myelinated axons, whereas only 3/6 animals of the DC-HX showed positive results (Fig. 4).

### Evaluation of short-term regeneration

**Fig. 5:**
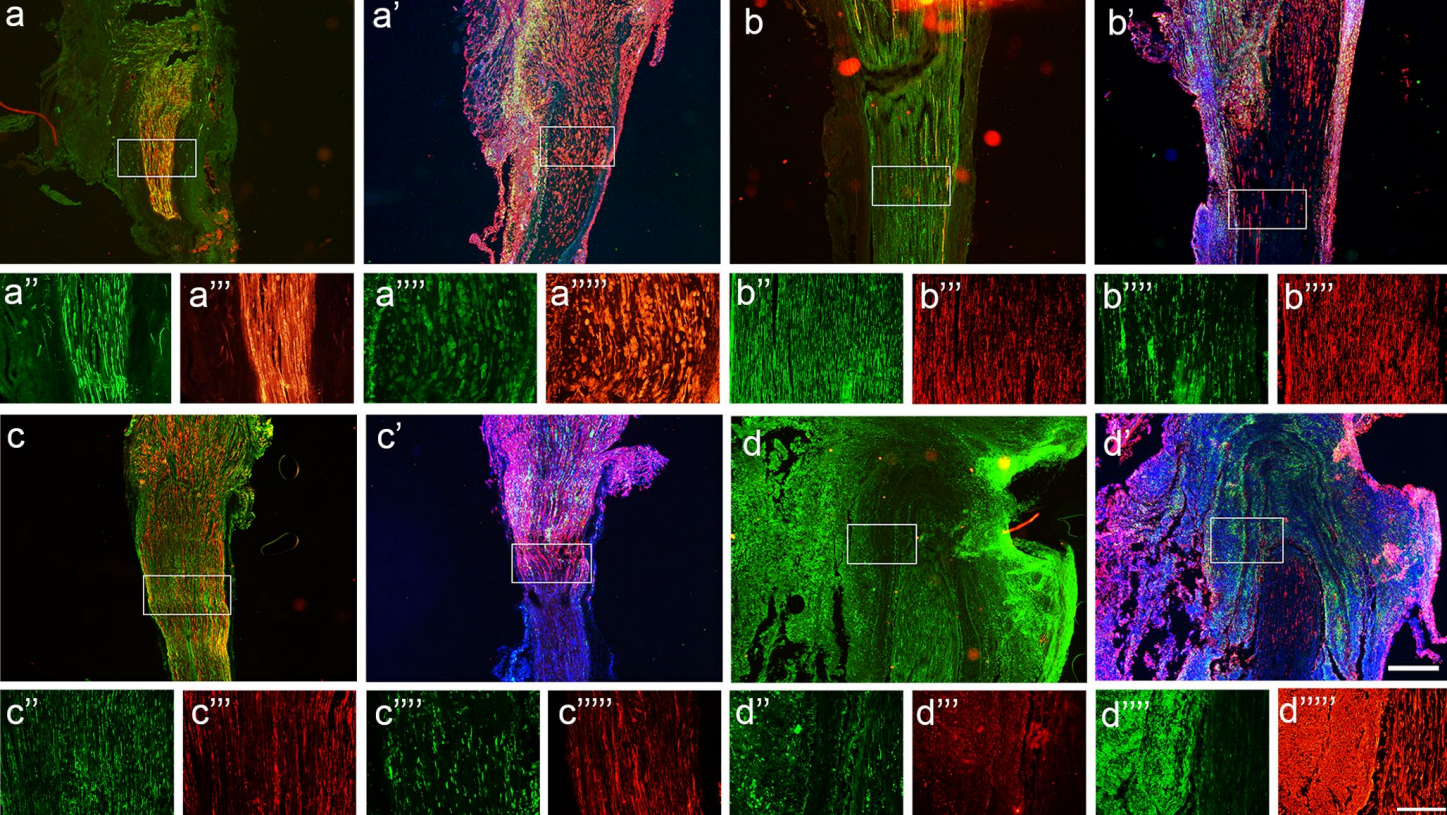
Immunohistochemical characterization of the grafts 1 and 2 weeks after implantation. Representative micrographs showing immunofluorescence of proximal segment of DC-RA at 7 days post injury (**a‴″**) labeling myelinated axons and Schwann cells (**a**), and macrophages, fibroblasts and DAPI (**a′**). Proximal segment of DC-HX at 7 days post injury (**b‴″**) labeling myelinated axons and Schwann cells (**b**), and macrophages, fibroblasts, and DAPI (**b′**). Proximal segment of DC-RA at 14 days post injury (**c‴″**) labeling myelinated axons and Schwann cells (**c**), and macrophages, fibroblasts, and DAPI (**c′**). Proximal segment of DC-HX at 14 days post injury (**d‴″**) labeling myelinated axons and Schwann cells (**d**), and macrophages, fibroblasts and DAPI (d′). Images (**a**, **a′**, **b**, **b′**, **c**, **c′**, **d**, **d′**) taken at 40 × magnification; scale bar 500 µm. Below images showed myelinated axons labeled against NF200 (**a″**, **b″**, **c″**, **d″**), Schwann cells labeled against S100 (**a‴**, **b‴**, **c‴**, **d‴**), macrophages labeled against Iba1 (**a″″**, **b″″**, **c″″**, **d″″**), fibroblasts labeled against vimentin (**a‴″**, **b‴″**, **c‴″**, **d‴″**), images taken at 200 × magnification; scale bar 200 µm

Considering the above results, a short-term study was performed to assess the early events of the regenerating nerves in the two types of decellularized grafts. At 7 dpi, the axonal regenerative front, labeled with NF200, reached the middle of the graft in rats of both groups, although there was higher number of axons in DC-RA than in DC-HX grafts (Fig. 5a). There were relatively few Schwann cells accompanying the regenerating axons. In the DC-RA group, marked proliferation of fibroblasts was observed within the nerve, whereas in the DC-HX group, the fibroblast invasion was mainly around the nerve. The Iba1 reactivity, corresponding to macrophages, was higher in the DC-HX group (Fig. 5b).

In samples taken at 14 dpi, a greater number of Schwann cells compared to 7 dpi was present around regenerating axons (Fig. 5c), whereas fibroblasts showed a more organized longitudinal organization. In the DC-HX group, fibroblasts were present both inside and outside the graft, indicating an important fibrotic reaction, whereas macrophages were only present around the nerve (Fig. 5d). Besides, only one of the three animals presented regenerated axons along the nerve graft. In the DC-RA group, the regenerative front reached the distal segment of the graft. In the DC-HX group, regenerating axons reached the distal segment in the only animal that had already NF positive axons at the proximal segment. As expected, in the other two animals, the graft was disorganized, with infiltrating cells and marked presence of macrophages. This is in contrast to what we observed at 7 days, when all animals of the group had some regenerative axons in the proximal half of the graft. It is possible that the important fibrotic reaction developed at 14 dpi overcame the regenerative response observed at early time points.

### Influence of ECM origin on neurite growth of DRG neurons in culture

**Fig. 6:**
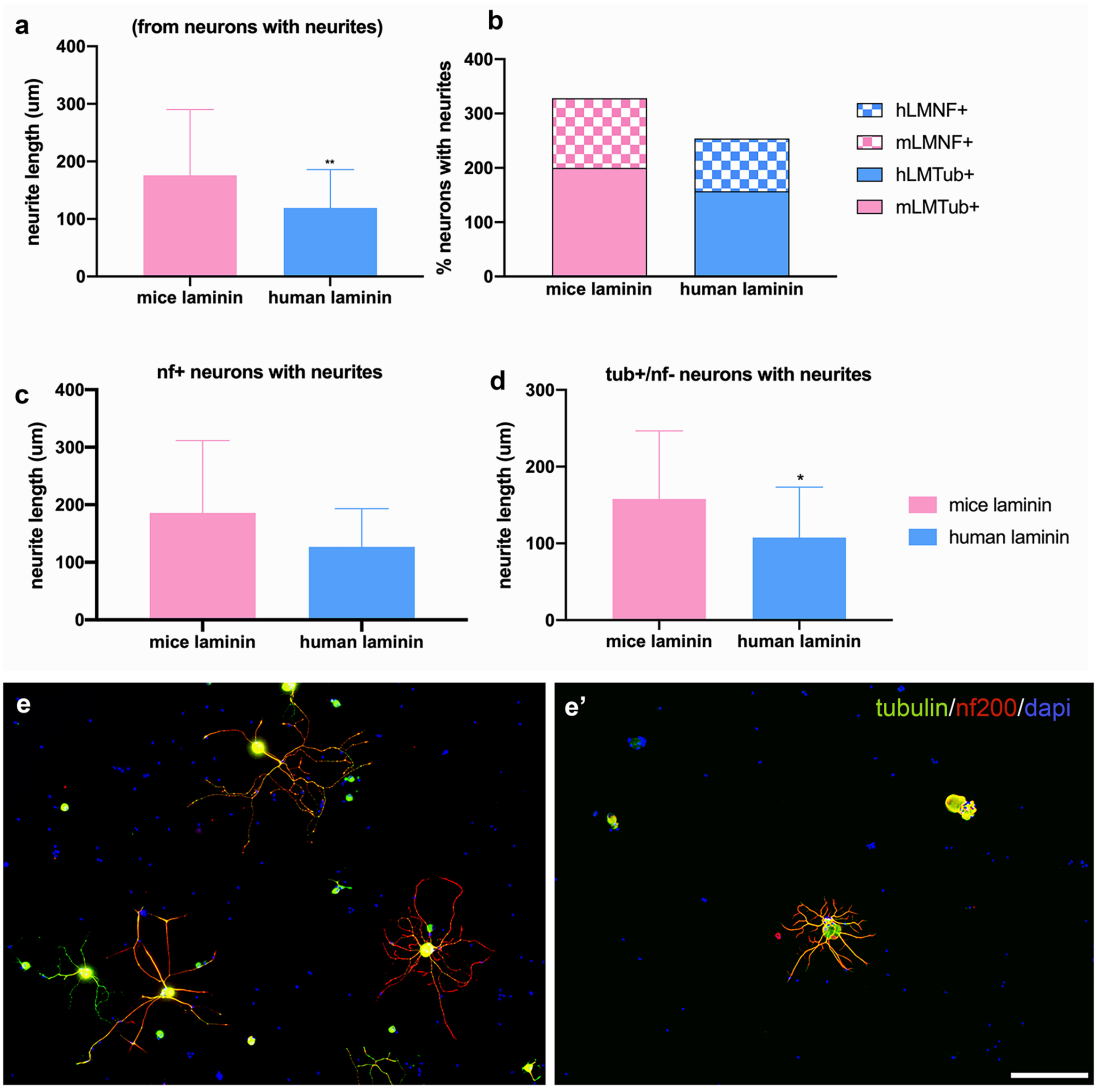
In vitro neural growth on laminins from different species. Plots showing the the neurite length of all neurons (**a**), the percentage of neurons with neurites (**b**), the neurite length of myelinated neurons (nf200 +) (**c**) and non-myelinated (nf200 −) neurons with neurites (**d**). *p < 0.5 vs mice laminin; **p < 0.01 vs mice laminin. Mouse DRG neuronal culture immunolabeled against β3-tubulin (green), nf200 (red) and dapi (blue), on surface coated with mouse laminin (**e**) or with human laminin (**e′**). Higher number of neurites and longer neurites can be observed on mouse laminin than on human laminin. Images taken at 100 × magnification; scale bar 200 µm

To evaluate if inter-species differences in ECM components, specifically in laminin, could play a role in the poorer ability of decellularized xenografts to sustain axonal regeneration, we compared neurite growth of mice DRG neurons on slides coated with laminin either from human or from mouse origin. When mice neurons were platted on mice laminin, a higher number of neurons with neurites were observed compared to human laminin. Mean of neurite length from neurons with neurites was significantly higher in mouse laminin (175.7 ± 114.4 µm) compared to human laminin (118.9 ± 66.9 µm, *p* < 0.0001). When analyzing neurite growth of myelinated (NF positive) and non-myelinated (NF negative) neurons, similar results were found. Therefore, mean neurite length of NF positive neurons with neurites (185.7 ± 125.9 µm and 126.7 ± 66.5 µm, *p* < 0.0001) and NF negative neurons with neurites (157.9 ± 88.7 µm and 106.28 ± 66.2 µm, *p* < 0.001) (Fig. 6) was significantly higher on mouse laminin compared to human laminin.

## Discussion

In this study, we have performed the first in vivo assay of a novel, optimized protocol for nerve decellularization that fulfills the standardized recommendations for peripheral nerve decellularization (Nieto-Nicolau et al. 2021), and demonstrated that such decellularized allograft allows for effective axonal regeneration along a very long gap in the rat, paving the way to its translation to the clinic. We have also shown that a decellularized xenograft is not adequate to ensure nerve regeneration due to interspecies differences of the ECM components to guide adequate migration of regenerating cells and axons.

Successful nerve regeneration requires an orchestrated series of events that implies a switch of neurons to a pro-regenerative state and important changes at the distal stump, that lead to Wallerian degeneration and the creation of a permissive milieu for axonal regeneration, where Schwann cells, immune cells, endothelial cells, and fibroblasts play an important role supporting axonal regrowth (Mcdonald et al. 2006; Allodi et al. 2012; Kim et al. 2013; Dun and Parkinson 2015; Cattin and Lloyd 2016; Roballo and Bushman 2019). Despite the potential of peripheral nerves to regenerate, successful functional recovery is usually limited after severe nerve lesions (Navarro et al. 2007). Thus, nerve resections that lead to long gaps between nerve stumps present poor success of regeneration and commonly limited recovery if surgically repaired (Pfister et al. 2011). This limitation is partially due to the lack of good repair alternatives to the gold standard autograft. Since it is mandatory to bridge the gap to guarantee some degree of regeneration, research has focused on a variety of graft substitutes, including decellularized graft (Nagao et al. 2011). A decellularized graft has to provide an off-the-shelf alternative to synthetic conduits while partially maintaining some of the proregenerative properties of autologous nerve grafts (Kasper et al. 2020). Currently, there is only one commercial cadaveric decellularized nerve allograft, Axogen Avance^®^ (AxoGen Inc., Alachua, FL, USA) approved for clinical application by the FDA (Kornfeld et al. 2019, 2021) but the limited evidence of its pro-regenerative potential has prevented its expansion to the European market. Therefore, further studies aimed to optimize protocols to decellularize human cadaveric nerves and to evaluate its regenerative potential compared to the gold standard autografts are needed. In this sense, decellularization is a key element when using allografts since the presence of cells on these grafts trigger a strong immune reaction in the recipient that limits their clinical use. The targets of rejection in peripheral nerves are mainly the Schwann cells and the myelin sheaths, because their membranes carry the antigens of the major histocompatibility complex (Evans et al. 1995). Systemic immunosuppression is effective for avoiding rejection (Udina et al. 2003), but it is not a convenient alternative for this type of non-vital graft, since it would place the patient at risk of infection, toxicity, and other complications (Szynkaruk et al. 2013; Vasudevan et al. 2014). Therefore, the decellularization protocol has to guarantee elimination of donor antigens while preserving the ECM and the physical architecture of the nerve, two elements that are fundamental to sustain successful regeneration. In this sense, our protocol of decellularization (Nieto-Nicolau et al. 2021) successfully preserves the ECM and endoneurial tubules, visualized by immunolabeling against laminin and collagen IV, while removing all types of cells, as demonstrated by the lack of DAPI and the Schwann cell marker S100 in both rat and human decellularized grafts.

More importantly, this optimized decellularized allograft allows successful axonal regeneration when used to repair a critical 15 mm nerve defect in the rat sciatic nerve. However, regeneration and reinnervation, evaluated by both electrophysiological tests and histological techniques, is slower in decellularized allografts compared to the native autografts. This finding is not surprising, since the decellularization eliminates all cellular components, among them Schwann cells, that are key elements orchestrating the regenerative response in the distal stump. Migration of host Schwann cells into the allograft is needed to create a permissive milieu for regeneration, a fact that explains the slower regeneration observed into a decellularized graft (Hall 1996).

On the other hand, regeneration and reinnervation were more limited when the same gap was repaired using a decellularized xenograft, obtained from a human nerve. As previously described (Wood et al. 2014), there were appreciable qualitative differences in the arrangement of axons (more disorganized) and in the density of myelinated axons (reduced) into the graft and distally in the decellularized xenografts compared to decellularized allografts and more with respect to the cellular autografts. A previous study also reported the higher pro-regenerative capability of allografts compared to xenografts, when repairing a smaller, 10 mm rat sciatic nerve defect (Kvist et al. 2011), whereas another found minimal differences by using the same gap to repair the rat facial nerve (Huang et al. 2015). The origin of the donor could hardly explain these differences, since Kvist et al. compared xenografts from different donors and could not found a simple relation between the origin of the graft and the extent to which it supports axonal outgrowth (Kvist et al. 2011).

It remains unclear why and how interspecies differences between the host and the donor graft affect nerve regeneration (Kvist et al. 2011; Wood et al. 2014). By using a decellularized graft, no immune rejection is expected. Therefore, we decided to evaluate and compare short-term regeneration within decellularized allografts and xenografts. Consistent with the long-term study, we found that regeneration was faster in DC-RA group, whereas in the DC-HX group was limited or even failed; furthermore, this failure was accompanied with a marked cellular infiltration, evident at two weeks after grafting, not earlier, suggesting that a period of time is necessary to trigger an inflammatory/rejection response in these grafts, negatively impacting the regenerative process.

Interestingly, in the DC-RA grafts, we observed an early fibroblast proliferation, that was organized in aligned tubes inside the nerve at 14 days post grafting. It is well established that fibroblasts are key players in the formation of the initial bridge between the two nerve stumps after nerve transection (Parrinello et al. 2010). This bridge will be later invaded by migrating Schwann cells to create the adequate milieu for axons to regenerate through the graft. In fact, migration of Schwann cells is directed by fibroblasts and endothelial cells (Cattin et al. 2015), whereas Schwann cell invasion precede growth of the axons through the bridge (Chen et al. 2005; Mcdonald et al. 2006; Parrinello et al. 2010). In the case of a decellularized graft, both fibroblasts and Schwann cells have to migrate from the proximal and distal nerve stumps and re-cellularize the graft. Recellularization by Schwann cells seems mandatory to guarantee regeneration, but fibroblasts are also important to facilitate attraction of Schwann cells. However, when we analyzed the decellularized grafts at 7dpi, we observed that axons preceded Schwann cells, indicating that the decellularized graft already offers a permissive milieu for axonal regeneration, thanks to the preserved architecture and the presence of ECM components. In addition, fibroblasts aligned along the endoneurial tubules, would present a pro-regenerative profile that favor axonal regeneration and contrast with the fibrotic reaction observed in other situations, that is detrimental for regeneration. In fact, in DC-HX grafts, large number of fibroblasts were also observed, but mainly located around the nerve, in a reaction compatible with a foreign body response. Therefore, the different response of fibroblasts can contribute to the different regenerative potential of these two types of grafts.

On the other hand, the potential inter-species differences in ECM components can also contribute to the poorer regenerative potential of a xenograft compared to an allograft. Laminin is an important component of the ECM that interacts with axons and Schwann cells through binding with integrins (Gonzalez-Perez et al. 2013; Mckerracher et al. 1996) and that also promotes neurite growth in culture (Baron-Van Evercooren et al. 1982). We found that laminin was present in the basal lamina, inside the preserved endoneurial tubules, similarly in both types of decellularized grafts. However, when we compared the capability of mouse neurons to extend neurites in vitro, in wells coated with laminin either from mouse or from human sources, we observed that laminin from the same specie sustained stronger and longer neurite growth. This intra-specie preference can contribute to the higher potential of allografts to sustain axonal growth compared to xenografts (Kvist et al. 2011).

In conclusion, a nerve decellularization protocol that guarantees elimination of cellular contents preserving the structure and the ECM of the graft can become an alternative to autografts to repair long nerve defects, although regeneration is going to be slower in the decellularized grafts. In contrast, xenografts showed some limitations, mainly related to inter-species immune response, fibrotic reaction, and the lower capacity of ECM components to sustain axon growth of neurons from another specie. Our results demonstrate the translationality of the optimized protocol for nerve decellularization that fulfills the standardized recommendations (Nieto-Nicolau et al. 2021), and also its success as a decellularized allograft to sustain regeneration over long nerve gaps in rats.
