# Correction to: A novel decellularized nerve graft for repairing peripheral nerve long gap injury in the rat

**DOI:** 10.1007/s00441-022-03737-3

**Published:** 2023-01-18

**Authors:** Estefanía Contreras, Sara Bolívar, Núria Nieto‑Nicolau, Oscar Fariñas, Patrícia López‑Chicón, Xavier Navarro, Esther Udina

**Affiliations:** 1 Department of Cell Biology, Physiology and Immunology, Institute of Neuroscience, Universitat Autònoma de Barcelona, and CIBERNED, ISCIII, Bellaterra, 08913 Spain; 2 Barcelona Tissue Bank, Banc de Sang I Teixits (BST), Barcelona, Spain; 3 Biomedical Research Institute (IIB-Sant Pau; SGR1113), Barcelona, Spain

**Correction to: Cell and Tissue Research (2022) 390:355–366** **https://doi.org/10.1007/s00441-022-03682-1**

The authors regret that there was a mistake in the formula used to estimate density of regenerative axons in the samples for the “In vivo long-term studies”, that affect all the experimental groups, underestimating their real values. The text of the result section describing the histological findings (Histological evaluation, second paragraph) has to be as follows:

“Quantitative analysis demonstrated that the density of myelinated axons was statistically higher in AG (40,267.78 ± 2775.7 axons/mm^2^) than in the DC-RA (28,132.47 ± 3084.11 axons/mm^2^, *p < 0.5 vs AG) and DC-HX (18,244.98 ± 3070.41 axons/mm^2^, $*p* < 0.5 vs DC-RA and ***p < 0.001 vs AG) groups in the middle of the graft. Distal to the graft, the myelinated fiber density was also significantly higher in AG group (27,293.08 ± 195.8 axons/mm^2^) compared to DC-RA (14,337.13 ± 1324.54 axons/mm^2^, **p < 0.01) and DC-HX (2289.29 ± 513.73 axons/ mm^2^, #*p* < 0.01 vs DC-RA and ****p < 0.0001). All animals showed regeneration in the middle of the grafts. Distal to the nerve graft, all animals from AG and DC-RA had myelinated axons, whereas only 3/6 animals of the DC-HX showed positive results (Fig. 4).”

Figure 4 has been corrected accordingly, as well as figure legend. 

The sentence of the abstract refering to the histology section has also been  corrected. "The density of myelinated axons was significantly higher in AG compared to both DC grafts, being this density  significantly higher in DC-RA than in DC-HX."

**Fig. 4:**
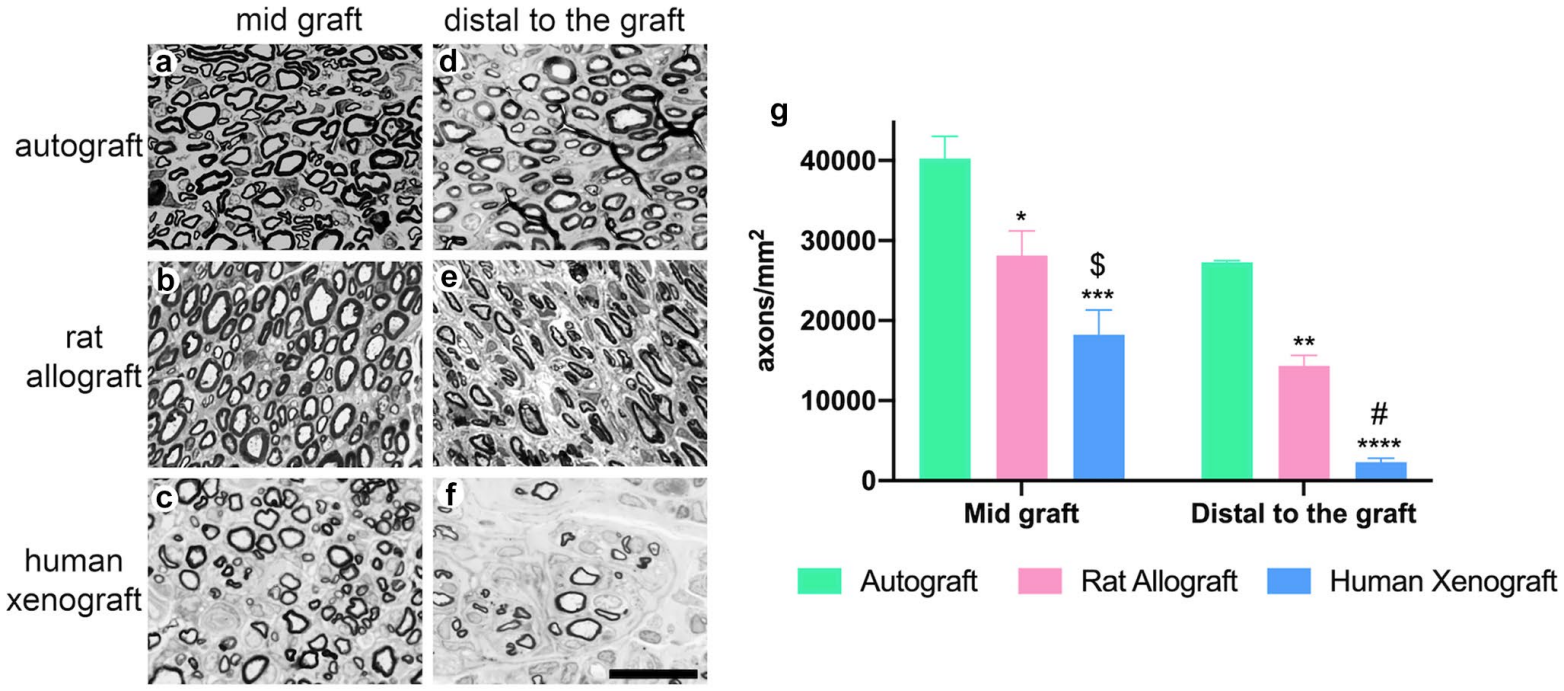
Histological evaluation of the regenerative potential of the grafts at 120 days. Representative transverse semithin sections of the mid graft (**a**, **b**, **c**) and distal to the graft (**d**, **e**, **f**) in autograft (**a**, **d**), decellularized rat allograft (**b**, **e**), and decellularized human xenograft (**c**, **f**) groups, stained with toluidine blue. Images were taken at × 1000 magnification; scale bar 10 μm. (**g**) Plots showing density of myelinated axons in the sciatic nerve at the mid graft and distal to the graft in the three groups. *p<0.5 vs AG; $*p*<0.5 vs DC-RA; **p<0.01 vs AG; #*p*<0.01 vs DC-RA; ***p<0.001 vs AG; ****p<0.0001 vs AG

It is now corrected in this erratum article.

The original article has been corrected.

